# Relationship between stress hyperglycemia ratio and allcause mortality in critically ill patients: Results from the MIMIC-IV database

**DOI:** 10.3389/fendo.2023.1111026

**Published:** 2023-04-03

**Authors:** Chong Zhang, He-Chen Shen, Wei-Ru Liang, Meng Ning, Zi-Xuan Wang, Yi Chen, Wei Su, Ting-Ting Guo, Kun Hu, Ying-Wu Liu

**Affiliations:** ^1^ The Third Central Clinical College of Tianjin Medical University, Tianjin, China; ^2^ Tianjin Key Laboratory of Extracorporeal Life Support for Critical Diseases, The Third Central Hospital of Tianjin, Tianjin, China; ^3^ Artificial Cell Engineering Technology Research Center, The Third Central Hospital of Tianjin, Tianjin, China; ^4^ Tianjin Institute of Hepatobiliary Disease, The Third Central Hospital of Tianjin, Tianjin, China; ^5^ Department of Heart Center, The Third Central Hospital of Tianjin, Tianjin, China; ^6^ State Key Laboratory of Experimental Hematology, National Clinical Research Center for Blood Diseases, Haihe Laboratory of Cell Ecosystem, Institute of Hematology & Blood Diseases Hospital, Chinese Academy of Medical Sciences & Peking Union Medical College, Tianjin, China

**Keywords:** all-cause mortality, critically ill patients, intensive care unit, stress hyperglycemia ratio, mortality

## Abstract

**Background:**

Stress hyperglycemia ratio (SHR) was developed to reduce the impact of long-term chronic glycemic factors on stress hyperglycemia levels, which have been linked to clinical adverse events. However, the relationship between SHR and the short- and long-term prognoses of intensive care unit (ICU) patients remains unclear.

**Methods:**

We retrospectively analyzed 3,887 ICU patients (cohort 1) whose initial fasting blood glucose and hemoglobin A1c data within 24 hours of admission were available and 3,636 ICU patients (cohort 2) who were followed-up for 1-year using the Medical Information Mart for Intensive Care IV v2.0 database. Patients were divided into two groups based on the optimal cutoff value of SHR, which was determined using the receiver operating characteristic (ROC) curve.

**Results:**

There were 176 ICU deaths in cohort 1 and 378 patients experienced all-cause mortality during 1 year of follow-up in cohort 2. The results of logistic regression revealed that SHR was associated with ICU death (odds ratio 2.92 [95% confidence interval 2.14–3.97] *P* < 0.001), and non-diabetic patients rather than diabetic patients showed an increased risk of ICU death. As per the Cox proportional hazards model, the high SHR group experienced a higher incidence of 1-year all-cause mortality (hazard ratio 1.55 [95% confidence interval 1.26–1.90] *P* < 0.001). Moreover, SHR had an incremental effect on various illness scores in predicting ICU all-cause mortality.

**Conclusion:**

SHR is linked to ICU death and 1-year all-cause mortality in critically ill patients, and it has an incremental predictive value in different illness scores. Moreover, we found that non-diabetic patients, rather than diabetic patients, showed an increased risk of all-cause mortality.

## Introduction

Patients suffering from trauma and critical illness often experience stress-induced hyperglycemia (SIH), even if they have no history of diabetes, leading to insulin resistance (IR) due to acute stress, inflammatory reaction, and severe disruption in glucose metabolism. Their glucose levels are often above average. During the period of sympathetic overactivity, catecholamine inhibits insulin release and promotes glycogen decomposition, which reduces the glucose uptake of the heart and leads to overactivity of hyperglycemia related proinflammatory pathway (1, 2). In acute myocardial infarction (AMI) patients, the SIH is a cause of over-inflammation and myocardial scar extension, which could over-activate inflammatory and fibrotic pathways with consequent worse clinical outcomes (3). Previous studies have demonstrated gut and thrombus microbiota dysbiosis and the miR33/sirtuin 1 pathway increased pro-inflammatory and pro-coagulable state of coronary thrombi, and thrombus aspiration could reduce clinical outcomes in AMI patients with SIH (4–6). Up to now, numerous studies have demonstrated that SIH is associated with adverse outcomes in the intensive care unit (ICU) for critically ill patients (1, 7–11).

However, in the absence of evidence, the glucose level was chosen arbitrarily. Therefore, the definition of the optimal cut-off value of SIH is inconsistent among guidelines. The European Society of Cardiology (ESC) recommends that SIH be defined as an admission blood glucose level higher than 11 mmol/L, whereas the American Heart Association (AHA) recommends that SIH be defined as an admission blood glucose level higher than 10 mmol/L, irrespective of diabetes diagnosis (12, 13). Currently, the optimal threshold of SIH and its association with the prognosis of ICU patients remain unclear and need to be addressed urgently.

In addition, it is necessary to include diabetic patients to comprehensively evaluate the prognosis of SIH in ICU patients. The results of a previous study were inconclusive about the prognostic value of SIH in diabetic patients (1), which suggests that SIH may not be a reliable risk factor in some special cases. To avoid misestimating the true prevalence of stress hyperglycemia, the stress hyperglycemia ratio (SHR) was developed to reduce the impact of long-term chronic glycemic factors on stress hyperglycemia levels (14). Moreover, previous studies mainly focused on SHR and clinical adverse outcomes in AMI, whereas few studies focused on ICU patients.

In this study, we evaluated the cut-off values of SHR in predicting ICU all-cause mortality and 1-year all-cause mortality in critically ill patients. In addition, we included SHR in evaluating the predictive value of various risk scores.

## Methods

### Study population

This is a retrospective study, and data were extracted from the Medical Information Mart for Intensive Care IV v2.0 (MIMIC-IV-v2.0) database. MIMIC-IV-v2.0 is a single-center database that was updated based on MIMIC-IV-v1.0. It was released on June 12, 2022 and is maintained by the Massachusetts Institute of Technology (MIT). The database contains medical data from Beth Israel Deaconess Medical Center (Boston, Massachusetts, USA) from 2008 to 2019. One of the authors (Hechen Shen) is authorized to use the database (Record ID 49784899).

**Figure 1:**
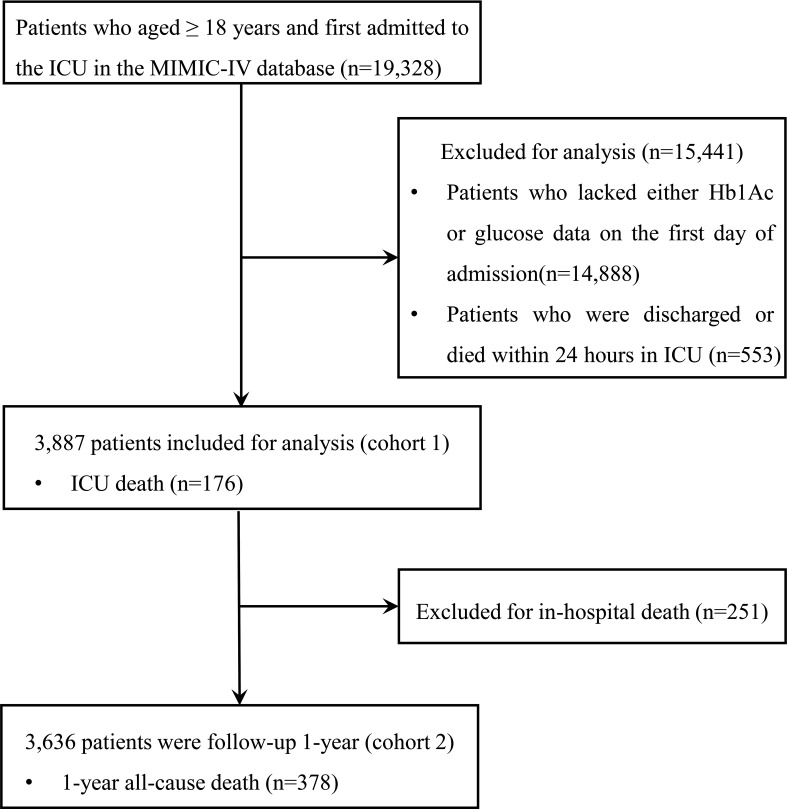
Flow chart of the study. ICU, intensive care unit.

We included 19,328 patients in our analysis. Patients admitted to the ICU for the first time and aged ≥ 18 years were included. The exclusion criteria included: (1) patients aged < 18 years; (2) patients whose HbA1c or fasting blood glucose data within 24 hours of admission were unavailable; and (3) patients who were discharged or died within 24 hours in the ICU. Finally, a total of 3,887 patients were included in the cohort (cohort 1) and divided into two groups according to the cutoff values for SHR. Moreover, 3,636 patients who were discharged from the hospital were followed up for 1 year (cohort 2). The flow chart is shown in **Figure 1**.

### Study variables

Data related to hospitalization within 24 hours of ICU admission were extracted from the MIMIC-IV-v2.0 database using PostgreSQL (version 14.5) and Navicat Premium (version 15.0) software. The data included the following variables: body mass index (BMI), gender, age, first ICU type, heart rate, diastolic blood pressure (DBP), systolic blood pressure (SBP), pulse blood oxygen saturation (SPO_2_), hemoglobin, white blood cells, platelets, glucose, hemoglobin a1c (HbA1c), creatinine, sequential organ failure assessment (SOFA) score, systemic inflammatory response syndrome (SIRS) score, logistic organ dysfunction system (LODS) score, acute physiology score III (APS III), model for end-stage liver disease (MELD), and oxford acute severity score (OASIS). SHR was calculated as [(admission glucose (mg/dl))/(28.7×HbA1c (%)-46.7)] (14). Comorbidities refer to coronary heart disease, hypertension, diabetes, chronic obstructive pulmonary disease (COPD), and chronic kidney disease (CKD), prediabetes. The diagnosis of comorbidities was determined based on the discharge diagnosis according to the documented International Classification of Diseases, version 9 (ICD-9) codes and International Classification of Diseases, version 10 (ICD-10) codes.

### Adverse clinical events

The primary outcomes included ICU all-cause mortality and 1-year all-cause mortality. The secondary outcomes included length of stay (LOS) in the ICU. Survival time is determined by date of death in the MIMIC-IV-v2.0 database.

### Statistical analysis

Patients in the two cohorts were divided into two groups based on the optimal cut-off value of SHR (< 1.10 or ≥ 1.10) and the optimal cutoff value of SHR (< 1.18 or ≥ 1.18), respectively, which was calculated using the receiver operating characteristic (ROC) curve. Continuous variables with a normal distribution are expressed as mean ± standard deviation (SD). Nonparametric continuous variables are expressed as the medians and interquartile ranges (25^th^–75^th^). Categorical variables are expressed as frequencies and percentages. The unpaired t test, Mann–Whitney U test, and chi-square test were used to test for differences between groups for continuous and categorical variables. Multivariate imputation (MI) was used to handle missing values less than 30%. Covariates that have been demonstrated the traditional risk factors for mortality were used for the adjustment model included age, gender, hypertension, diabetes, COPD, and CKD. Logistic regression, multiple linear regression, and sensitivity analysis were used to estimate the ICU adverse outcomes associated with SHR in cohort 1. By adding SHR to the severity of the illness scores (SIRS score, SOFA score, MELD score, APSIII score, LODS score, OASIS score), the area under the curve (AUC) was used to assess the accuracy of predicting ICU all-cause mortality. Kaplan–Meier survival analysis and Cox proportional hazards models were used to estimate the 1-year percentage of cumulative survival in cohort 2. We used the Cox proportional hazards models to estimate the relationship between SHR and 1-year all-cause mortality among subgroups. We used Stata 17.0 (Stata Corp, College Station, TX) for all analyses. A two-tailed P value < 0.05 was considered statistically significant.

## Results

### Baseline characteristics

Finally, 3,887 ICU patients were included in cohort 1 and 3,636 patients who were followed up for 1 year after discharge from the hospital were included in cohort 2. There were 176 patients who experienced ICU death and 251 patients who experienced in-hospital all-cause mortality. During the 1-year follow-up, 378 patients experienced all-cause mortality. The optimal cut-off value of SHR to predict ICU all-cause mortality was 1.23 (sensitivity 0.59, specificity 0.68, Youden’s index = 0.27, AUC=0.63) and that to predict 1-year all-cause mortality was 1.18 (sensitivity 0.47, specificity 0.65, Youden’s index = 0.12, AUC=0.56). Patients in the high SHR group were more likely to have higher glucose levels, higher severity of illness scores, suffer from hypertension, prolonged ICU LOS, and a higher incidence of ICU all-cause mortality compared with the low SHR group (**Table 1**). As shown in **Table S1**, patients in the high SHR group were more likely to have higher severity of illness scores, higher glucose levels, higher HbA1c levels, comorbidities, and a higher incidence of 1-year all-cause mortality. Although prediabetes leads to coronary endothelial dysfunction (15), there were no difference in prediabetes patients between the two groups among cohort 1 and cohort 2 in this study.

**Table 1:** Baseline characteristics and events of cohort 1.

Categories	Low SHR n=2604	High SHR n=1283	*P* value
Demographic			
Age, year	63.3 ± 14.8	63.1 ± 14.5	0.62
Sex, male, n (%)	1563 (60.0)	709 (55.3)	0.005
Body mass index, kg/m^2^	28.4 ± 5.3	28.4 ± 5.6	0.75
Comorbidities			
CHD, n (%)	1142 (43.9)	428 (33.4)	<0.001
CKD, n (%)	500 (19.2)	284 (22.1)	0.032
COPD, n (%)	32 (1.2)	27 (2.1)	0.036
Hypertension, n (%)	804 (30.9)	306 (23.9)	<0.001
Prediabetes, n (%)	29 (1.1)	9 (0.7)	0.22
Diabetes, n (%)	1073 (41.2)	607 (47.3)	<0.001
Laboratory tests			
Creatinine, μmol/L	0.9 (0.7, 1.3)	1.1 (0.8, 1.6)	<0.001
Glucose, mmol/L	115.0 (99.0, 133.0)	189.0 (156.0, 255.0)	<0.001
HbA1c	5.9 (5.5, 6.8)	5.8 (5.3, 6.6)	<0.001
LDL, mg/dL	81.0 (72.0, 93.0)	81.0 (73.0, 82.0)	0.016
ALT, U/L	27.0 (25.0, 27.0)	27.0 (24.0, 46.0)	<0.001
AST, U/L	38.0 (33.0, 38.0)	38.0 (35.0, 72.0)	<0.001
HGB, mg/dL	11.5 (10.0, 13.1)	11.5 (9.8, 13.3)	0.72
Plt, K/μL	210.0 (159.0, 270.0)	216.0 (163.0, 281.0)	0.055
WBC, K/μL	12.2 (9.0, 16.7)	13.7 (9.9, 18.1)	<0.001
ICU admission			
OASIS score	31.0 (25.0, 37.0)	34.0 (27.0, 41.0)	<0.001
LODS score	4.0 (2.0, 6.0)	5.0 (3.0, 8.0)	<0.001
APSIII score	40.0 (29.0, 54.0)	49.0 (35.0, 68.0)	<0.001
SIRS score	2.0 (2.0, 3.0)	3.0 (2.0, 3.0)	<0.001
SOFA score	3.0 (1.0, 5.0)	3.0 (2.0, 6.0)	<0.001
MELD score	11.0 (8.0, 17.0)	13.0 (9.0, 20.9)	<0.001
First Care Unit			<0.001
CVICU	901 (34.6)	207 (16.1)	
CCU	383 (14.7)	250 (19.5)	
MICU/SICU	919 (35.3)	623 (48.6)	
NSICU	207 (7.9)	77 (6.0)	
TSICU	194 (7.5)	126 (9.8)	
Vital signs			
Heart rate, bmp	82.0 (71.0, 93.0)	84.0 (73.0, 96.0)	<0.001
SBP, mmHg	118.0 (111.0, 125.0)	118.0 (113.0, 126.0)	0.25
DBP, mmHg	61.0 (57.0, 65.0)	61.0 (57.0, 65.0)	0.99
SpO_2_, %	97.0 (96.0, 98.0)	97.0 (96.0, 98.0)	0.67
Events			
LOS ICU, days	2.7 (1.6, 5.1)	3.9 (2.1, 7.6)	<0.001
ICU death, n (%)	74 (2.8)	102 (8.0)	<0.001

SHR, stress hyperglycemia ratio; CHD, coronary heart disease; CKD, chronic kidney disease; COPD, chronic obstructive pulmonary disease; HbA1c, hemoglobin a1c; LDL, low density lipoprotein; ALT, alanine transaminase; AST, aspartate aminotransferase; HGB, hemoglobin; WBC, white blood cell; ICU, intensive care unit; SOFA, sequential organ failure assessment; LODS, logistic organ dysfunction system; SIRS, systemic inflammatory response syndrome; OASIS, Oxford acute severity score; APS III, acute physiology score III; MELD, model for end-stage liver disease; CCU, coronary care unit; CVICU, cardiovascular intensive care unit; MICU/SICU, medical intensive care unit/surgical intensive care unit; TSICU, trauma/surgical intensive care unit; NSICU, neurosurgical intensive care unit.

### Association between ICU prognosis and SHR

Logistic regression revealed the association between SHR (≥ 1.23) and ICU death both in the crude model (odds ratio [OR] 2.95 [95% confidence interval (CI) 2.17–4.01] P < 0.001) and the adjusted model (OR 2.92 [95% CI 2.14–3.97] *P* < 0.001). In sensitivity analysis, non-diabetic patients rather than diabetic patients showed an increased risk of ICU death (**Table 2**). In addition, the multiple linear regression model showed SHR was found as a significant predictor of ICU LOS (**Table S2**).

**Table 2:** Association between the ICU death and SHR.

Categories	Crude model		Adjust model	
	OR and 95%CI	*P* value	OR and 95%CI	*P* value
ICU death				
Low SHR	Ref.		Ref.	
High SHR	2.95 (2.17 to 4.01)	<0.001	2.92 (2.14 to 3.97)	<0.001
ICU death (diabetic patients)				
Low SHR	Ref.		Ref.	
High SHR	1.87 (1.14 to 3.07)	0.014	1.80 (1.09 to 2.97)	0.021
ICU death (non-diabetic patients)				
Low SHR	Ref.		Ref.	
High SHR	4.03 (2.71 to 5.98)	<0.001	3.90 (2.62 to 5.80)	<0.001

SHR, stress hyperglycemia ratio; ICU, intensive care unit; OR, odds ratio; CI, confidence interval.

### Association between SHR and 1-year all-cause mortality

**Figure 2:**
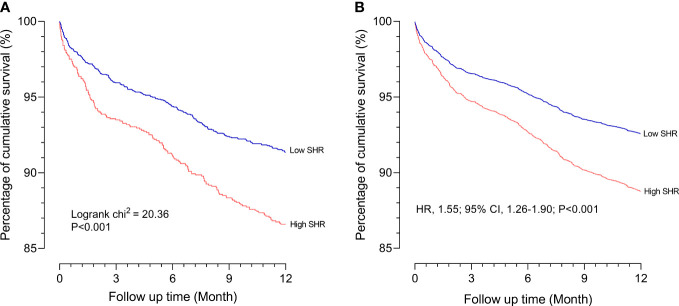
Association between the 1-year all-cause mortality and SHR **(A)** Kaplan-Meier survival analysis curves for all-cause mortality according to low SHR group and high SHR group at 1 year; **(B)** Survival curves according to low SHR group and high SHR group in the Cox proportional hazards model after adjustment for age, gender, hypertension, diabetes, COPD, and CKD. SHR, stress hyperglycemia ratio; COPD, chronic obstructive pulmonary disease; CKD, chronic kidney diseases.

**Figure 3:**
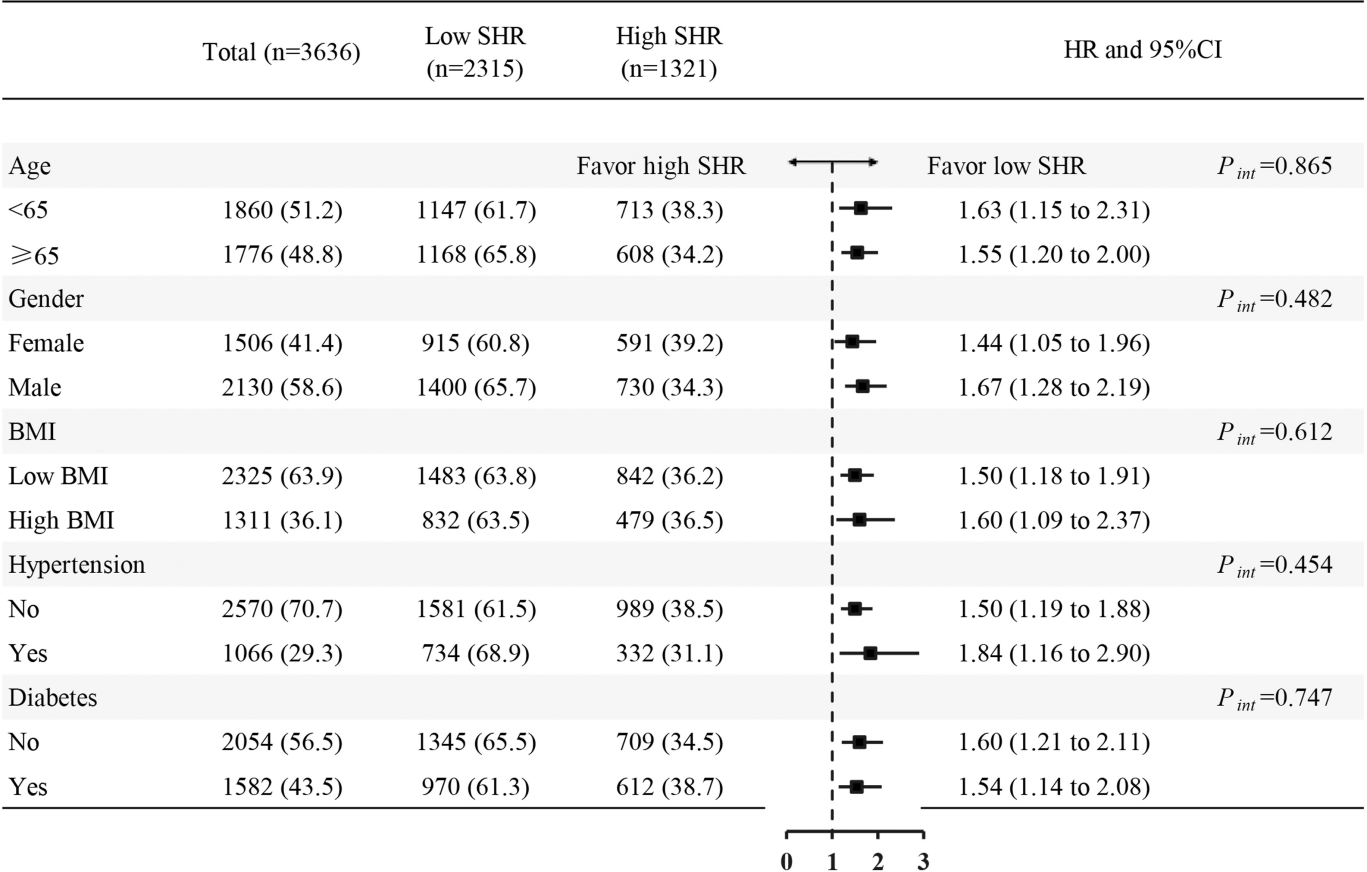
Forest plots of hazard ratios for the 1-year all-cause mortality in different subgroups. HR, hazard ratio; CI, confidence interval; SHR, stress hyperglycemia ratio; BMI, body mass index.

Cox proportional hazards models showed a significant association between SHR (≥ 1.18) and 1-year all-cause mortality both in the crude model (hazard ratio [HR] 1.59 [95% CI 1.30–1.94] P < 0.001) and the adjusted model (HR 1.55 [95% CI 1.26–1.90] *P* < 0.001). Kaplan–Meier survival analysis and Cox proportional hazards models revealed that the high SHR group had a higher incidence of 1-year all-cause mortality than the low SHR group (**Figure 2**). In addition, we estimated the relationship between SHR (≥ 1.18) and 1-year all-cause mortality among subgroups, including BMI, age, gender, diabetes, and hypertension. SHR was associated with increased risk of 1-year all-cause mortality in the subgroups of age < 65 years [HR 1.63 (95% CI 1.15–2.31)], male [HR 1.67 (95% CI 1.28–2.19)], BMI ≥ 30 kg/m^2^ [HR 1.60 (95% CI 1.09–2.37)], hypertension [HR 1.84 (95% CI 1.16–2.90)], and non-diabetic [HR 1.60 (95% CI 1.21–2.11)] (**Figure 3**).

### Incremental effect of SHR on ICU death

SHR had an incremental effect on the AUC of various illness scores in predicting ICU death, including SIRS score (0.639 vs. 0.689, *P* < 0.001), SOFA score (0.688 vs. 0.719, *P* = 0.004), MELD score (0.600 vs. 0.686, *P* < 0.001), APSIII score (0.788 vs. 0.804, *P* = 0.004), LODS score (0.781 vs. 0.802, *P* = 0.001), and OASIS score (0.788 vs. 0.801, *P* = 0.016) (**Table 3**).

**Table 3:** Incremental effect of SHR in predicting ICU all-cause death.

Categories	Model 1	Model 2	*P* value (Model 2 vs. Model 1)
	AUC and 95%CI	AUC and 95%CI	
ICU death			
SIRS Score	0.639 (0.600 to 0.678)	0.689 (0.647 to 0.730)	<0.001
SOFA Score	0.688 (0.646 to 0.730)	0.719 (0.678 to 0.760)	0.003
MELD Score	0.600 (0.558 to 0.643)	0.686 (0.649 to 0.724)	<0.001
APSIII Score	0.788 (0.754 to 0.821)	0.804 (0.773 to 0.836)	0.004
LODS Score	0.781 (0.744 to 0.818)	0.802 (0.768 to 0.836)	0.001
OASIS Score	0.788 (0.759 to 0.816)	0.801 (0.772 to 0.829)	0.016

SHR, stress hyperglycemia ratio; ICU, intensive care unit; SOFA, sequential organ failure assessment; LODS, logistic organ dysfunction system; SIRS, systemic inflammatory response syndrome; OASIS, Oxford acute severity score; APS III, acute physiology score III; MELD, model for end-stage liver disease; AUC, area under the curve; CI, confidence interval; Model 1, various illness scores; Model 2, various illness scores+SHR.

## Discussion

In this study, we explored the relationship between SHR and ICU patients prognosis and found that high SHR was associated with ICU death and long-term all-cause mortality. The cut-off values of SHR in predicting ICU death and 1-year all-cause mortality were 1.23 and 1.18, respectively. Moreover, we found an incremental effect of SHR as a novel and efficient biomarker for various illness scores in predicting ICU all-cause mortality.

Numerous studies have shown that ICU patients with SIH have an increased risk of worsening clinical outcomes (1, 7–11). SIH usually occurs in ICU patients and increases evidently within 48 hours in at least 50% of patients (16). SIH can increase IR and glucose levels in the body, which can cause the release of catecholamines, cortisol, glucagon, and growth hormones, promoting inflammation (2). In addition, ICU patients are often on parenteral nutrition with increased fat content, hyperglycaemia and inflammatory cytokines, such as interleukin-1 (IL-1), tumor necrosis factor-α (TNF-α), NAPDH oxidase-2 (NOX2) and NAPDH oxidase-1 (NOX1), which disrupts the body’s metabolic pathways and leads to worsening clinical outcomes (17–19). Studies have found that SIH is associated with ICU death. Mamtani et al. (20) showed SIH was related to ICU death and a longer length of ICU stay in 739,152 non-diabetic patients. Rau et al. (21) included patients with trauma and found SIH was associated with a higher injury severity score. SHR can eliminate the interference of long-term chronic glycemic factors on stress hyperglycemia levels, which can reflect the stress response in the body. Previous studies found that SHR had a prognostic value in AMI (1, 22, 23) and acute ischemic stroke patients (24, 25). However, there are few studies focused on ICU patients. Our study further provides clinical evidence to support the notion that SHR is associated with adverse events in ICU patients.

Moreover, SHR had an incremental effect on the predictive value of various illness scores in predicting ICU all-cause mortality. These illness scores have been demonstrated to have a predictive value in predicting adverse outcomes in ICU patients (26–29). Considering that SHR is essential to the prognosis of ICU patients and is an easily available, reliable, and inexpensive test, we innovatively added SHR to various illness scores and found the predictive value for ICU all-cause death was significantly improved.

Notably, in the subgroup analysis, we also compared the association between SHR and 1-year all-cause mortality under various risk factor stratifications. Specifically, we found that non-diabetic patients had a higher risk of 1-year all-cause mortality than diabetic patients, which is consistent with the ICU deaths among non-diabetic patients and diabetic patients in the sensitivity analysis. Current studies have demonstrated that non-diabetic patients, rather than diabetic patients, have a higher risk of adverse events (21, 30). We consider the following mechanisms to explain this finding: Firstly, diabetes is an inflammatory clinical entity with different mechanisms involved in its physiopathology (31). However, in the course of diabetes, diabetic patients are adapted to chronic oxidative stress and consistently higher hyperglycemia levels than non-diabetic patients. Moreover, diabetic patients who have been treated with insulin, which has a better anti-inflammatory effect (32–34).

Our study demonstrated the translational potential role of SHR in predicting ICU death and 1-year all-cause mortality in critically ill patients. However, there are several limitations to this study. Firstly, this was an observational study. Although we utilized careful and rigorous statistical methods to control for bias, further studies should be performed to explore the relationship between SHR and the prognosis of ICU patients. Secondly, the data was derived from the United States, where people have different physical conditions and lifestyles from the East Asian population. Thirdly, due to patients’ previous medical information about previous myocardial infarction, history of percutaneous coronary intervention (PCI), history of coronary artery bypass grafting (CABG) and pre-hospital medication, that cannot be obtained from the MIMIC-IV-v2.0 database, we cannot effectively address this bias. Prospective studies are needed to further investigate the effect of this bias on clinical outcomes.

## Conclusions

SHR has translational potential in predicting ICU all-cause mortality and 1-year all-cause mortality in critically ill patients, with incremental predictive value in various illness scores. Moreover, we found that non-diabetic patients, rather than diabetic patients, had a higher risk of ICU all-cause mortality and 1-year all-cause mortality.

## Data availability statement

The original contributions presented in the study are included in the article/**Supplementary Material**. Further inquiries can be directed to the corresponding author.

## Ethics statement

All procedures performed in studies involving human participants were in accordance with the ethical standards of the institutional and/or national research committee and with the 1964 Helsinki declaration. The use of the MIMIC-IV-v2.0 database was approved by the review committee of Massachusetts Institute of Technology and Beth Israel Deaconess Medical Center. The data is publicly available (in theMIMIC-IV-v2.0 database) hence ethical approval statement and the informed consent is not required for the study.

## Author contributions

Conception and design of the research: CZ, H-CS, W-RL, Y-WL, MN. Acquisition of data: H-CS, W-RL. Analysis and interpretation of the data: CZ, H-CS, W-RL, Z-XW, MN, YC, WS, T-TG, KH, Y-WL. Statistical analysis: CZ, H-CS, W-RL, Z-XW, YC, WS, T-TG, KH. Obtaining financing: Y-WL. Writing of the manuscript: CZ, H-CS, W-RL, Z-XW. Critical revision of the manuscript for intellectual content: MN, YC, WS, T-TG, KH, Y-WL. Moreover, CZ, H-CS, W-RL, MN have contributed equally to this work. All authors read and approved the final draft.

## Supplementary material

Supplementary Table 1 Baseline characteristics and events of cohort 2. SHR, stress hyperglycemia ratio; CHD, coronary heart disease; CKD, chronic kidney disease; COPD, chronic obstructive pulmonary disease; HbA1c, hemoglobin a1c; LDL, low density lipoprotein; ALT, alanine transaminase; AST, aspartate aminotransferase; HGB, hemoglobin; WBC, white blood cell; ICU, intensive care unit; SOFA, sequential organ failure assessment; LODS, logistic organ dysfunction system; SIRS, systemic inflammatory response syndrome; OASIS, Oxford acute severity score; APS III, acute physiology score III; MELD, model for end-stage liver disease; CCU, coronary care unit; CVICU, cardiovascular intensive care unit; MICU/SICU, medical intensive care unit/surgical intensive care unit; TSICU, trauma/surgical intensive care unit; NSICU, neurosurgical intensive care unit.

Supplementary Table 2 The association between SHR and ICU LOS. SHR, stress hyperglycemia ratio; LOS, length of stay; CKD, chronic kidney disease; COPD, chronic obstructive pulmonary disease; Adjustment model included age, gender, hypertension, diabetes, COPD, CKD.
