# Supplementary material for: Relationship between stress hyperglycemia ratio and allcause mortality in critically ill patients: Results from the MIMIC-IV database
Source: Front Endocrinol (Lausanne). 2023 Apr 3;14:1111026. doi: 10.3389/fendo.2023.1111026 (PMC10106677; doi:10.3389/fendo.2023.1111026)
Supplement: Supplementary Table 1 — Baseline characteristics and events of cohort 2. SHR, stress hyperglycemia ratio; CHD, coronary heart disease; CKD, chronic kidney disease; COPD, chronic obstructive pulmonary disease; HbA1c, hemoglobin a1c; LDL, low density lipoprotein; ALT, alanine transaminase; AST, aspartate aminotransferase; HGB, hemoglobin; WBC, white blood cell; ICU, intensive care unit; SOFA, sequential organ failure assessment; LODS, logistic organ dysfunction system; SIRS, systemic inflammatory response syndrome; OASIS, Oxford acute severity score; APS III, acute physiology score III; MELD, model for end-stage liver disease; CCU, coronary care unit; CVICU, cardiovascular intensive care unit; MICU/SICU, medical intensive care unit/surgical intensive care unit; TSICU, trauma/surgical intensive care unit; NSICU, neurosurgical intensive care unit. [file Table_1.docx]

**Supplemental Material**

**List of Content**

1. **Supplemental Tables**

- **Table S1.** Baseline characteristics and events of cohort 2
- **Table S2**. The association between SHR and ICU LOS

 **Table S1.** Baseline characteristics and events of cohort 2

| Categories | Low SHR n=2315 | High SHR n=1321 | *P* value |
| --- | --- | --- | --- |
| Demographic |  |  |  |
| Age, year | 63.3±14.6 | 62.8±14.3 | 0.31 |
| Sex, male, n (%) | 1400 (60.5) | 730 (55.3) | 0.002 |
| Body mass index, kg/m^2^ | 28.5±5.3 | 28.5±5.6 | 0.97 |
| Comorbidities |  |  |  |
| CHD, n (%) | 1047 (45.2) | 448 (33.9) | <0.001 |
| CKD, n (%) | 442 (19.1) | 293 (22.2) | 0.026 |
| COPD, n (%) | 28 (1.2) | 29 (2.2) | 0.021 |
| Hypertension, n (%) | 734 (31.7) | 332 (25.1) | <0.001 |
| Prediabetes, n (%) | 27 (1.2) | 10 (0.8) | 0.24 |
| Diabetes, n (%) | 970 (41.9) | 612 (46.3) | 0.010 |
| Laboratory tests |  |  |  |
| Creatinine, μmol/L | 0.9 (0.7, 1.3) | 1.0 (0.8, 1.6) | <0.001 |
| Glucose, mmol/L | 113.0 (98.0, 130.0) | 180.0 (148.0, 244.0) | <0.001 |
| HbA1c | 5.9 (5.6, 6.8) | 5.8 (5.3, 6.5) | <0.001 |
| LDL, mg/dL | 81.0 (71.0, 93.0) | 81.0 (73.0, 88.0) | 0.140 |
| ALT, U/L | 27.0 (25.0, 27.0) | 27.0 (25.0, 39.0) | <0.001 |
| AST, U/L | 38.0 (33.0, 38.0) | 38.0 (35.0, 61.0) | <0.001 |
| HGB, mg/dL | 11.5 (10.0, 13.0) | 11.5 (9.8, 13.2) | 0.33 |
| Plt, K/μL | 210.0 (159.0, 269.0) | 215.0 (163.0, 283.0) | 0.034 |
| WBC, K/μL | 11.9 (8.9, 16.4) | 13.3 (9.7, 17.7) | <0.001 |
| ICU admission |  |  |  |
| OASIS score | 30.0 (25.0, 37.0) | 33.0 (26.0, 40.0) | <0.001 |
| LODS score | 4.0 (2.0, 6.0) | 5.0 (3.0, 7.0) | <0.001 |
| APSIII score | 39.0 (29.0, 52.0) | 46.0 (34.0, 64.0) | <0.001 |
| SIRS score | 2.0 (2.0, 3.0) | 3.0 (2.0, 3.0) | <0.001 |
| SOFA score | 3.0 (1.0, 5.0) | 3.0 (2.0, 5.0) | <0.001 |
| MELD score | 11.0 (8.0, 16.6) | 13.0 (8.0, 20.1) | <0.001 |
| First Care Unit |  |  | <0.001 |
| CVICU | 840 (36.3) | 245 (18.5) |  |
| CCU | 336 (14.5) | 240 (18.2) |  |
| MICU/SICU | 799 (34.5) | 643 (48.7) |  |
| NSICU | 179 (7.7) | 63 (4.8) |  |
| TSICU | 161 (7.0) | 130 (9.8) |  |
| Vital signs |  |  |  |
| Heart rate, bmp | 81.0 (71.0, 93.0) | 84.0 (74.0, 96.0) | <0.001 |
| SBP, mmHg | 118.0 (111.0, 124.0) | 118.0 (113.0, 126.0) | 0.14 |
| DBP, mmHg | 61.0 (57.0, 65.0) | 61.0 (58.0, 66.0) | 0.45 |
| SpO_2_, % | 97.0 (96.0, 98.0) | 97.0 (96.0, 98.0) | 0.92 |
| Events |  |  |  |
| 1-year all-cause death, n (%) | 201 (8.7) | 177 (13.4) | <0.001 |

Abbreviations: SHR=stress hyperglycemia ratio; CHD= coronary heart disease; CKD= chronic kidney disease; COPD=chronic obstructive pulmonary disease; HbA1c=hemoglobin a1c; LDL= low density lipoprotein; ALT=alanine transaminase; AST=aspartate aminotransferase; HGB= hemoglobin; WBC=white blood cell; ICU=intensive care unit; SOFA=sequential organ failure assessment; LODS=logistic organ dysfunction system; SIRS=systemic inflammatory response syndrome; OASIS=Oxford acute severity score; APS III=acute physiology score III; MELD=model for end-stage liver disease; CCU=coronary care unit; CVICU=cardiovascular intensive care unit; MICU/SICU=medical intensive care unit/ surgical intensive care unit; TSICU=trauma/surgical intensive care unit; NSICU=neurosurgical intensive care unit.

**Table S2.** The association between SHR and ICU LOS

| Categories | Crude model | |  | | Adjust model | |
| --- | --- | --- | --- | --- | --- | --- |
|  | Coef. and 95%CI | *P* value |  | | Coef. and 95%CI | *P* value |
| LOS hospital |  |  |  |  |  |  |
| SHR | 1.85 (1.45 to 2.26) | <0.001 |  |  | 1.75 (1.35 to 2.15) | <0.001 |

Abbreviations: SHR=stress hyperglycemia ratio; LOS=length of stay; CKD= chronic kidney disease; COPD=chronic obstructive pulmonary disease; Adjustment model included age, gender, hypertension, diabetes, COPD, CKD.
